# Effect of 808 nm Semiconductor Laser on the Stability of Orthodontic Micro-Implants: A Split-Mouth Study

**DOI:** 10.3390/ma13102265

**Published:** 2020-05-14

**Authors:** Jacek Matys, Rafał Flieger, Tomasz Gedrange, Krzysztof Janowicz, Bartosz Kempisty, Kinga Grzech-Leśniak, Marzena Dominiak

**Affiliations:** 1 Laser Laboratory at Dental Surgery Department, Medical University of Wroclaw, 50-425 Wrocław, Poland; kgl@periocare.pl; 2 Private Dental Practice, 64-000 Kościan, Poland; gabinet6@op.pl; 3 Dental Surgery Department, Medical University of Wroclaw, 50-425 Wrocław, Poland; tomgedrange@gmail.com (T.G.); marzena.dominiak@wp.pl (M.D.); 4 Department of Orthodontics, Technische Universität Dresden, Fetscherstr. 74, 01307 Dresden, Germany; 5 Department of Anatomy, Poznan University of Medical Sciences, 60-781 Poznan, Poland; krzysztof.janowicz.16@abdn.ac.uk (K.J.); bkempisty@ump.edu.pl (B.K.); 6 Department of Histology and Embryology, Poznań University of Medical Sciences, 60-781 Poznań, Poland; 7 Department of Obstetrics and Gynaecology, University Hospital and Masaryk University, 602 00 Brno, Czech Republic; 8 Department of Veterinary Surgery, Institute of Veterinary Medicine, Nicolaus Copernicus University, 87-100 Toruń, Poland

**Keywords:** laser biostimulation, low level laser therapy, mini-implant therapy, PBM, PTV

## Abstract

Background: To evaluate the effect of photobiomodulation (PBM) on orthodontic micro-implants (n = 44; 14 women, 8 men). Methods: PBM with 808 nm diode laser was applied immediately, 3, 6, 9, 12, 15, and 30 days post the implantation. Results were assessed within same time frames and additionally after 60 days to check for implants stability using the Periotest device. Patients pain experiences following the first day post-treatment and potential loss of micro-implants after 60 days were recorded. The procedure involved insertion of mini-implants in the maxilla for the laser group (L, n = 22) and negative control group (C, n = 22). Irradiation was carried buccally and palatally with respect to the maxillary ridge (2 points). The energy per point was 4 J (8 J/cm^2^), total dose was 56 J. Results: Patients did not report significant differences in terms of pain experiences comparing the L and C groups (*p* = 0.499). At 30 days post-treatment, higher secondary stability of implants was observed in the laser group (Periotest Test Value, PTV 6.32 ± 3.62), in contrast to the controls (PTV 11.34 ± 5.76) (*p* = 0.004). At 60 days post-treatment, significantly higher stability was recorded in the laser group (PTV 6.55 ± 4.66) compared with the controls, PTV (10.95 ± 4.77) (*p* = 0.009). *Conclusions:* Application of the 808 nm diode laser increased secondary micro-implant stability.

## 1. Introduction

Due to implications concerning the use of traditional anchoring methods, including anchoring instability, micro-implants (micro-screws, mini-implants) have appeared as a novel and more efficient strategy [1,2]. The high chances of micro-implant losses followed by surgery have recently been overcome by orthodontic micro-implants, which are increasingly used in therapies [3]. Stabilization at the beginning of the treatment is crucial to improving the total efficiency of implant-based therapies, further supplemented by mechanical retention [4,5]. Even though maintaining osseointegration is not required, implants are incorporated just after surgery [6]. Consideration of volume and quality of bone are recognized as critical aspects for successful osteointegration and continuous clinical success of the treatment [7]. In addition, measuring the implant’s stability and bone density is necessary for regenerative dentistry and mini-implant associated orthodontics [7]. While the biomechanical stability (primary stability) of implants allows their immediate loading, it mainly depends on the state of the bone-implant interface [6,7,8]. Importantly, other aspects contributing to implant stability include their surface morphology and chosen surgical strategy [9]. The two most commonly used tests to assess implant stability are the Periotest, and resonance frequency analysis (RFA) due to their having the least invasiveness [7,8,9], with Periotest used mainly for measuring primary stability. The procedure is based on pistil incorporated into the Periotest device, which is accelerated, followed by its deflection in accordance with the periodontal status of implant or tooth [10]. Damping properties of the peri-implant tissue are qualified in a numerical system (−8 to +50) with lower values indicating higher implant stability [11]. At the cellular level, PBM is also known as low-level laser therapy (LLLT) and is mainly used to improve the non-thermal photochemistry effect prescribed by Adenosine Triphosphate (ATP) synthesis, resulting in enhanced primary stability of mini-implants and bone healing properties [12,13]. Low-energy doses are used to produce added ATP, so in fact, LLLT excites the mitochondrial photoreceptors, resulting in improved proliferation and differentiation of cells, including the osteoblasts [14,15,16]. Near-infrared laser application applied in different studies delivered positive outcomes in terms of implant contact factor (BIC) [17,18]. LLLT was demonstrated to trigger mitosis during in vivo cell culture resulting in nucleic acids and collagen synthesis [15]. LLLT was further investigated to improve the regeneration and healing of fractured tissues and nerve revitalization processes [19]. In the nearest future, PBM still needs to be probed in terms of biochemical stability of orthodontic mini-implants [9,20,21,22]. 

Different action mechanisms describe the effect of PBM (LLLT) at the cellular level, most importantly including change in metabolomics of cell due to a photochemical impact [23]. As soon as the portion of laser energy is transported to mitochondrion, the enzyme cytochrome C oxidase (COX) absorbs the photons [24]. Following this process, changes in mitochondrial respiratory chains result in an increase in the synthesis of ATP, nitric oxide (NO), calcium ions, and reactive oxygen species (ROS) [25]. The products of irradiation chemical reactions (e.g., NO, ROS) serve as signaling molecules, activating multiple metabolic processes in cells [26]. Nitric oxide absorbed by oxidase cytochrome C increases the proton gradient, which results in increases in ATP, ROS, and calcium ions production [27,28]. The following processes enhance various cell differentiation of fibroblasts, osteoblasts and chondrocytes among other cell types. Laser energy absorbed by COX evokes the ROS activation phenomenon [29]. Reactive Oxygen Species in cells activate many cellular metabolic pathways, e.g., p38 mitogen-activated protein kinases (p38 MAPK) and protein kinase D2 (PRKD2) [28]. Just as p38 MAPK and PRKD2 pathways increase hormone activity of melatonin, the same is true for differentiation of osteoblasts [30]. The effect of PBM at the cellular level is dependent on the energy dose and the laser light wavelength [24]. The study by Wang et al. indicates the positive impact of 810 nm wavelength absorbed by COX mitochondrial activity [31]. Another study states that optimal results can be evoked with PBM at a wavelength of 810 nm [32]. This is coincident with the wavelength (808 nm) used in the present research. In the present study, the effects of PBM on osteoblasts differentiation were investigated. Our literature review (Pubmed, Google Scholar) indicates only a few studies examining the influence of PBM on orthodontic micro-screws stability. Five of these studies were in the animal [9,18,20,33,34] and three in the human model [35,36,37]. Our research team recently published results of a split-mouth trial [7] assessing the stability of the mini-implants in patients receiving PBM with the red laser light at a wavelength of 635 nm at higher exposure radiation (20 J/cm^2^) in contrast to this present investigation. The aim of this research was to identify the clinically applicable influence of an 808 nm near-infrared semiconductor laser on orthodontic mini-screws stability, further confirmed in terms of patient-reported pain experiences on the first day after the treatment as well as a loss of micro-implants after 60 days.

## 2. Materials and Methods

### 2.1. Patients

**Figure 1:**
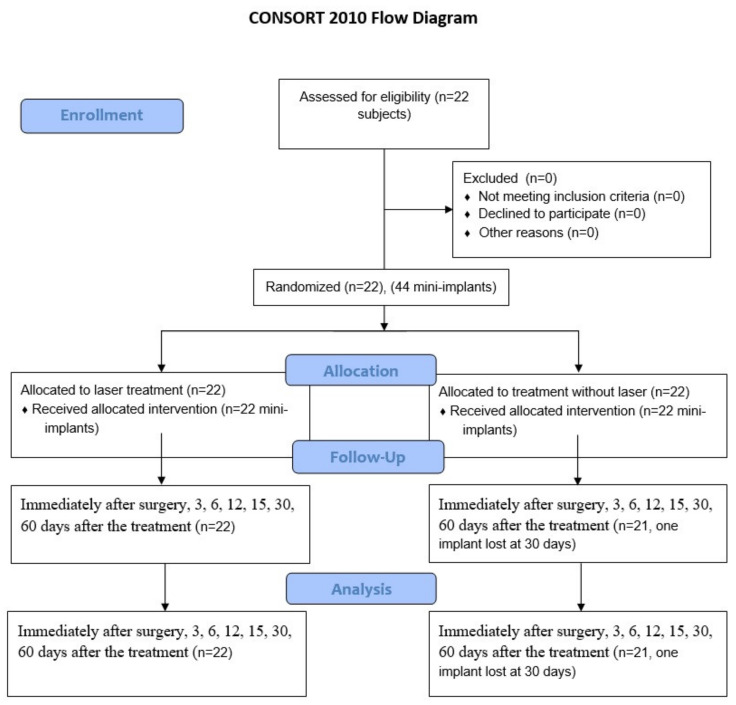
CONSORT 2010 flow diagram used in the research to help to assess for eligibility of subjects.

The implantation and irradiation were prepared and conducted as a randomized and controlled trial (RCT). The trial was registered at the official website ClinicalTrials.gov (identifier: NCT04276402). The approval of the Local Ethics Committee of Wrocław Medical University, Faculty of Dentistry was obtained (permission number: KB-278/2018), and informed consent in accordance with the Helsinki Declaration was obtained from all participating subjects. Before the study, each patient had a radiographic examination using orthopantomogram and lateral cephalometry. The protocol was applied for 44 orthodontic micro-implants inserted in the distal region of the maxilla. At the time of the study, the patients included 14 women and 8 men with a mean age of 31.7 ± 9.7 years (Figure 1); standard parameters, including significance level of 0.05, d value of 0.55, 95% confidence interval, and 80% power of the study, were used to calculate a sample size equal to 22 for each side of the maxilla using G×Power software, version 3.1. (Kiel University, Kiel, Germany) [6]. Only the most experienced implantologist conducted and supported the trials at Private Dental Healthcare in Kościan, Poland. Subjects with malocclusion (class II) defects requiring lateral maxillary teeth distalization were accordingly only selected for orthodontic implantation. Patients were non-smokers and investigated in the context of systemic diseases, uncompensated diabetes, or uncontrolled periodontal disease, and only those without signs of previous complications were further selected. Patients were not allowed to have used antibiotics or bisphosphonate medication during the previous two years. At first, prior to the clinical trial, subjects underwent hygienist treatment. Importantly, patients reported no history of radiotherapy reported treatment involved fixed orthodontic appliance without anti-inflammatory drugs.

### 2.2. Orthodontic Treatment, Surgical Procedures and Laser Irradiation

**Figure 2:**
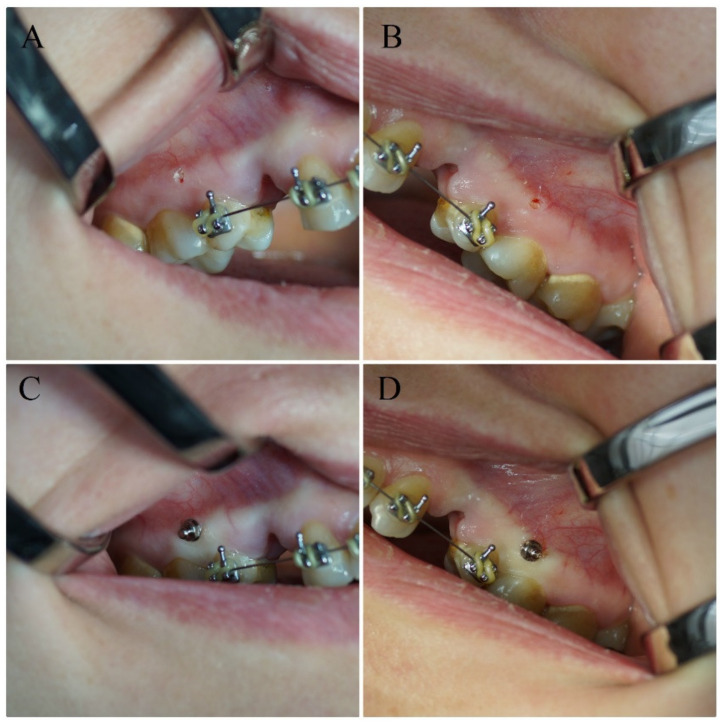
Soft tissue removal and implantation on the right (**A**,**C**) and left (**B**,**D**) side of the maxillary ridge.

**Figure 3:**
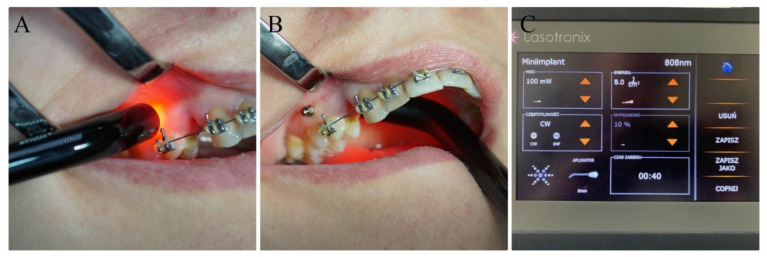
LLLT with the 808 nm laser buccally (**A**) and palatally (**B**) to the maxillary arch. (**C**) The 808 nm semiconductor laser (SmartM PRO, Lasotronix, Poland).

A fixed orthodontic appliance with MBT 022 (GC Orthodontics America, Alsip, IL, USA) prescription and a straight wire method were applied to subjects as a method for qualification for the clinical trials. The soft tissue was detached using a dental porcelain bur, followed by insertion of the implant in association with a hand driver. Implantation was conducted with respect to maintaining no bone decortications (Figure 2). A number of titanium 5th grade micro-implants 1.4 mm in width and 10 mm in length (RMO, West Colfax Ave, Denver, CO, USA) were placed at both sides of the maxillary arch among the first molar and the second premolar, two millimeters below the mucogingival junction. Another round of mouthwash was applied after the procedure, including ten milliliters of 0.1% chlorhexidine (Eludril, Pierre Fabre, Boulogne, France) for thirty seconds three times a day for three weeks. A coin toss was done to randomly identify the side of laser application in the maxilla. The laser (L, n = 22) and control (C, n = 22) group involved mini-implants inserted in the maxilla. Irradiation was carried buccally and palatally to the maxillary ridge (two points). Due to contact with peri-implant soft tissue, the settings of the laser were adjusted to output power equal to 100 mW. The energy per point was 4 J (8 J/cm^2^) at a constant pace of 40 s per point, two applications per session, dose in session 8 J. The 808 nm diode laser (SmartM PRO, Lasotronix, Warsow, Poland) at 808 nm wavelength was used with the following set parameters; output power: 100 mW; handpiece diameter: 8 mm; spot size: 0.5 cm^2^; average irradiance: 200 mW/cm^2^. The protocol of diode laser application: (L group) immediately and 3, 6, 9, 12, 15, 30 days later. Conducting all curative trials required an energy dose of 56 J (Figure 3). Implants were inserted on the opposite side of the maxilla in the controls (C group).

### 2.3. Measurement of Implants’ Stability, Pain Value and Calculation of Statistical Analysis

Measurement of implant stability occurred just after the surgery and three, six, nine, twelve, fifteen, thirty, and sixty days after the implantation. Following the mentioned periods, the measurements were repeated five times, and mean values were calculated, compared and assessed. The Periotest tool (Medzintechnik Gulden, Modautal, Germany) was used to measure implant stability. Measurements were carried out with respect to the soundwave signal received as a mean of contact between metallic tapping bar in a handpiece and the implant. FFT algorithm was used to analyze the received signal, supplemented by an accelerometer used to record and interpret the Periotest result. The Periotest value (PTV) is the result of the transformation of the signal to a value depending on the oscillation of stored energy dissipated by the damping of the peri-implant tissue [8]. Periotest value ranges from −8 to +50, originally prescribed by a mathematical algorithm. The higher the implant stability, the lower the Periotest value, and consequently, the higher the absorption wavelength of the target objects. The bar mechanism of work relies on electromagnetic trigger further verified electronically. Just after the surgery and insertion of mini-implants, all patients were asked to answer a questionnaire to assess individual pain levels 0–10 (the numeric rating scale NRS-11; Legend: 0 = no pain, 1–3 = little pain, 4–6 = modest pain, 7–10 = severe pain). Pain levels reaching ten were assessed at both sides of the maxilla during the first day after the treatment. Importantly, the results obtained using the NRS-11 scale were assessed on individuals older than ten years old.

### 2.4. Statistical Analysis

Assuming significance level of *p* = 0.05, Student’s *t*-test for dependent samples was used to check for differences in the pain value and micro-implant stability of the samples using the Statistica, version 12, StatSoft, Tulsa, OK, USA). The Kolmogorov–Smirnov test was performed at the 95% level of confidence and statistical analysis was performed to check the normal distribution of data. 

## 3. Results

**Figure 4:**
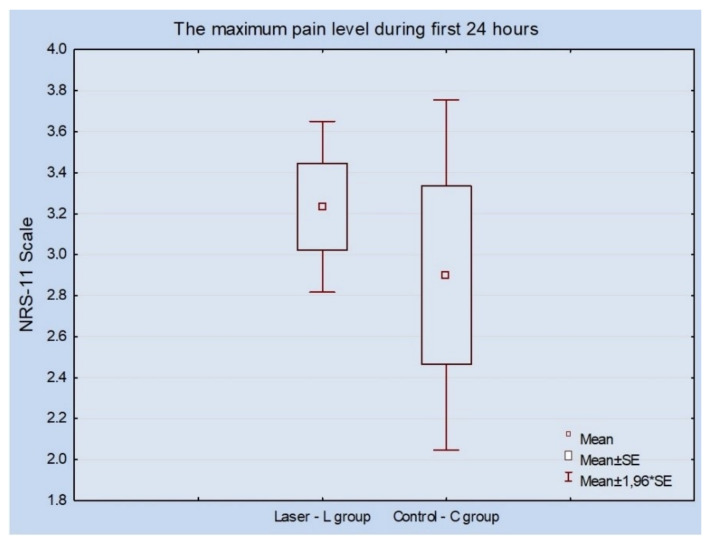
The highest pain score was achieved at 24 h post micro-implant placement.

One micro-implant was lost in the control group during the 60-day examination frame. The implant was removed on the last day of the experiment, and the orthodontic treatment for this patient was continued without additional anchoring on the micro-screw. No significant changes measured at either of the maxilla were reported by patients in the context of experienced pain (*p* = 0.499). The pain values at the laser side were in a shorter range as compared to the non-irradiated side (Figure 4).

Following the 60-day period, the overall results demonstrated an increase in the secondary stability of self-drilling micro-implants. Controls (C group: 10.95 ± 4.77) showed a higher mean value of PTV compared to the test group (L group: 6.55 ± 4.66) (*p* = 0.009). After 30 days, non-irradiated micro-implants showed a decrease in stability (PTV 11.34 ± 5.76) compared to the 808 nm laser-treated test group (PTV 6.32 ± 3.62) (*p* = 0.004). Importantly, the mean baseline value of PTV was similar at both sides and the difference in stability decrease of micro-screws was insignificant during the first two weeks. (Table 1) 

## 4. Discussion

PBM includes therapeutic approaches concerning bone healing [38], reduced pain in orthodontics [39], increased peri-implant bone density [40], regeneration and proliferation of fibroblasts and osteoblasts [16,38], and regeneration of neurons [19]. The main aim of the study was to probe the effects of PBM on stability of dental mini-implants placed in the maxilla. The failure rate and pain level were measured during the treatment. Specifically, the study involved the effect on implant stability when irradiated with 808 nm laser beam at 8 J/cm^2^ (4 J) per point. No RCT human split-mouth study has evaluated the role of PBM at 808 nm on the stability of micro-screws in orthodontics. The results indicated increased secondary stability using irradiation with 808 nm diode laser after 30 and 60 days. Only one mini-implant was lost on the control side in one subject during the 60 days. No significant differences in pain level were found between the test and control sides of the maxillary arch. These results were considered with reference to previous applications of LLLT (doses of four and sixteen Joules) in the context of lowering pain following elastomeric separator placement [41]. 

The pain threshold was recorded on the left and right sides of the maxillary arch, referring to previous findings of our other randomized clinical split-mouth study [7]. The first clinical trial concerning pain level assessment in a similar randomized study aimed to indicate higher forces as a main trigger for experiencing more severe pain by subjects [39], by evaluating pain level through exerting various forces. The higher the adjusted force was, the more deformed the orthodontic wire was, indicating that elastic strain may potentially influence the properties of the orthodontic wire. One of the aims of this study was to measure the effect of the PBM on pain level assessed just after implantation. No significant changes in pain level were recorded on either side of the maxilla. Similar results were found by our group for mini-implants irradiated with a diode laser at a wavelength of 635 nm and a dose per point of 10 J. In other recent literature, no consistent hypothesis was evaluated in the context of patient experienced pain during the orthodontic treatment. A concise analysis indicated that no existing data could be found in the literature in the context of the investigation being carried out. Considering two different laser energy values 4 and 16 J, split-mouth clinical studies showed no difference from the gold standard pioneer study [41] in terms of relieving elastomeric separators triggering orthodontic pain. Importantly, it is a fixed orthodontic appliance, which could also influence pain analysis [19]. Subjects reported their first pain experiences 2 to 4 h after the application of the force and the fixing of the orthodontic appliance. The pain was maintained over the first 24 h, progressively disappearing over the following seven days [19,41]. 

There are limitations concerning the clinical assessment of micro-screw stability and the effects of PBM. In a recently conducted clinical study [37], levels of peri-implant crevicular fluid during the 3 days following implantation were assessed. Yanaguizawa et al. reported elevated levels of interleukin IL-8 after 24 h, 48 h, and 72 h for non-irradiated patients. These findings suggest the anti-inflammatory activity of PBM in the context of irradiation at 660 nm wavelength and at 40 mW, for a duration of 1 min (on average, 2.4 J). Yanaguizawa further explained that the factors that influence the stability of the mini-implants increase following irradiation therapy. Another study by Osman et al. found an improvement in micro-implants stability [36] at 910 nm laser wavelength with an average power not exceeding 0.7 W for duration of 60 s. Ekizer et al. [35] delivered similar results using a light-emitting diode at an irradiance of 20 mW/cm^2^ after two and three months, respectively. In this study, 2 months post treatment, a rise in the stability of the micro-implants was also recorded, but for a diode laser at 635 nm wavelength at 100 mW, 20 J/cm^2^. Interestingly, in the studies by Osman at al. [36] and the previously conducted trials [7] (including the current study), differences in the stability of the mini-implants between the first and second months were insignificant. Analogously, differences in stability scores in the study by Ekizer et al. [35] similarly recorded between the second and third months were also non-significant. Therefore, the healing process is most efficient during the first week post treatment, especially during the inflammation phase. Application of PBM at the beginning and during the first phase of peri-implant bone healing holds the potential to modulate healing processes and consequently lead to greater micro-implant stability a month or two after.

The effects of LLLT/PPM are statistically dependent on laser light dose. Arndt-Schultz’s curve was used to estimate the LLLT effect, which was directly dependent on the energy dose. The advanced penetration depth of the LLLT spectrum sits in the optical window of 600 to 1100 nm, leading to the stronger signal of the light to cell reaction [16]. The results presented graphically as Arndt-Schultz’s curve suggested that a low stimulus could increase physiologic activity and regulate stimulus disruption activity. In the case of strong stimuli, total disruption and lack of activity were recorded [15,16]. At the third day after implementation, with adjustments detected in the peri-implant area, naturally occurring healing processes commenced, including provisional matrix development, granulation, tissue formation, blood clotting, and fiber and mesenchymal cell formation [42]. Consequently, fibroplasia and angiogenesis processes lasted three days (4–7 days following the surgery), and this is known as provisional connective tissue formation. Furthermore, evoked by placing the implants after the injury, the effect of the 808 nm wavelength on the inflammation of the bone was measured. During the first two weeks, irradiated mini-implants demonstrated a slower reduction pace in the context of implant stability than the controls [16]. Synthesis of woven bone was detected after 7 days, indicating the beginning of osseointegration [42]. Following the mini-implants insertion, the 808 nm laser was used five times over fifteen days due to the possibility of variation in the infrared laser beam, but mainly to alleviate and correct all steps occurring as inflammation begins [16]. 

The near-infrared wavelength at fluence between 1 and 10 J/cm^2^ is optimal to gain an ideal cellular response [16]. In this current investigation, the radiant exposure of 4 J (8 J/cm^2^) provided increased secondary mini-implant stability. Consequently, a higher energy dose was employed per session, and a broader window of optical range was established (1–10 J/cm^2^) [9,20,43]. Moreover, other approaches in the context of the irradiation effects on implant stability demonstrated increased freshly formed bone volume and non-significant changes in the context of Periotest value (0.65- to 0.79-fold lower). Looking for improvement in conducted studies, an analysis of other internationally recognized therapeutic trials was conducted [9,20]. Positive outcomes of LLLT in micro-screw stability had been proved on an animal model, reporting that the 808 nm semiconductor laser at an energy dose of five Joules increased the average pull-out strength compared to controls [8]. Our previously published study proved that a red laser at ten Joules per point improved the micro-implants stability [7]. In the present study, the energy dose per point (4 J) was lower in contrast to that used previously; however, the infrared wavelength (808 nm) had a deeper penetration [44] than the 635 nm diode laser; thus, the laser energy can be transported deeper into the tissue than the red laser and trigger the healing of injured tissues.

In total, it took 60 days to assess mini-implant loss over the 60 days. As mentioned previously, the conducted trial involved implantation of 44 implants, from which all remained and served as an anchorage method in the orthodontics. In vitro studies conducted beforehand demonstrated that the implant fracture is not favored when implants are implemented without bone decortication [11]. Additionally, working with a self-drilling system, the small diameter of the implant and the great initial stability leads to an elevated risk of implant deterioration [11]. To gain higher primary stability, all orthodontic micro-screws were placed without decortication of the bone ridge. The thickness of the mandibular arch is higher than that of the cortical lamina in the maxilla. Therefore, minimization of the forces needed to insert the implants can be accomplished by perforating cortical lamina of the bone with a density of over 800 HU (Hounsfield Unit) [11,45]. Because bone densities are different for the maxilla and mandible, and even for their different segments, the results of implant stability measured with various techniques can be different with respect to laser application as well. Some studies have observed differences in mini-implant stability in this regard. A recent meta-analysis published by Casaña-Ruiz et al. [46] showed greater stability of mini-implants inserted in a mandible, in contrast to the maxillary arch (the odds ratio of 0.56). However, the maxillary location showed significantly lower failure rates than the mandibular [46]. The most important factor affecting the success of clinical orthodontic therapy seems to be high implant stability (high bone density). In this present study, we placed micro-screws in adult subjects (22–41 years), and thus in patients with matured bone in the peri-implant area. Additionally, taking into account the influence of bone density and root topography, the implants were inserted in a similar location (between the second premolar and the first molar). This reduced the bias of the study connected with possible differences in bone density between the implantation sides. In our present study, only one mini-implant was lost in the control group during the 60-day examination frame. The mini-implant was removed at 60 days due to its mobility (PTV = 50). It should be highlighted that in previously published research, no mini-implant had been lost. These findings also confirmed the results obtained by Casaña-Ruiz et al. [46] showing a lower failure rate for micro-implants placed in the maxilla.

Furthermore, a reduction in micro-screw stability was observed during the first fifteen days. However, the final PTV score (decrease of implant stability) was lower at irradiated sites in comparison to the controls. Our previously published in vitro study proved that the use of small-sized orthodontic micro-screws increases the risk of implant failure [11]. Moreover, surgical intervention using the mini-implants causes inflammation in the area of implant to bone interface. The inflammation phase lasted around 14 days. Inflammation involved many biological processes including, e.g., blood clot formation, fibroplasia angiogenesis, and the formation of the new woven bone (end of the second week) [47]. Following the inflammation phase, during the reparative phase, bone maturation begun, which lasted for up to five months post-treatment. Final bone maturation occurred during the remodeling phase [47]; thus, the risk of micro-screw failure decreased [7]. In the present study, all micro-screws were non-loaded for three months, and no implants were lost in either of the study groups. The trial was completed after 60 days post treatment, but loading of the mini-screws was conducted after this period. Hence, to answer the hypothesis of whether reduced time between implant placement and implant loading is crucial for the orthodontic healing process, it is crucial to consider the early loading of mini-implants, which demonstrates better stability [48,49]. Nonetheless, a previously conducted study involving animal models suggested no significant differences or histological findings between immediate loading and delayed loading [50]. Alternatively, Jeong et al. [51] observed orthodontic micro-screw failure after loading during the first 12 weeks. The failure percentages decreased according to loading time following insertion. The highest failure rate, equal to 13.56%, was recorded when implants were loaded four weeks after the insertion. In this study, all implants (excluding one in the control group, removed after 30 days) were loaded after two months. The failure rate described by Jeong et al. [51] for mini-implants loaded at two months post insertion decreased to 8.97%. In our study, the PTV at two months was recorded to be 6.55 ± 4.66 and 10.95 ± 4.77 for the 808 nm laser and control groups, respectively. Therefore, this indicates that the near-infrared laser photobiomodulation could significantly reduce the failure rate of the micro-screw-loaded implants for up to 8 weeks post-treatment. Long-term clinical benefits of using the LLLT system require additional study and dozens of mini-implants applied in various subjects over different time periods. Peri-implant bone tissue was used to evaluate the effect of different wavelengths, red and infrared, respectively; however, it requires larger study groups, and therefore more subjects and controls. Additionally, more randomized studies with placebo groups applying different methods for the implant stability measurement, e.g., resonance frequency analysis (RFA), reverse torque test (RTT), impact hammer method (Periotest), patient quality of life analysis and other essential factors, e.g., chewing side, bone density, alveolar bone thickness, and root morphology, are needed.

## 5. Conclusions

Application of the 808 nm diode laser on peri-implant soft tissue increased the secondary micro-implant stability after one and two months. The diode laser application had no significant effect on pain score. During the treatment, only one mini-implant was lost on the control side in one subject during the 60 days. PBM seems to have a positive effect on peri-implant bone healing. Clinically, LLLT could be useful in orthodontics in terms of obtaining suitable stability of micro-screws serving as an additional anchoring system.

## Figures and Tables

**Table 1:** Irradiation results recorded in the form of Periotest values (PTVs) at various time points in both the L and C groups.

Period	Laser (L Group) (n = 22)	Std	Control (C Group) (n = 21)	Std	*p*-Value
Baseline	−1.25	2.65	−1.08	2.34	0.824
3 days	−0.66	3.10	−1.19	2.49	0.503
6 days	−0.54	2.71	−0.94	2.41	0.576
9 days	−0,79	2.37	−0.83	1.43	0.949
12 days	−0.02	2.48	−0.38	0.97	0.554
15 days	1.81	3.20	1.00	0.66	0.279
30 days	6.32	3.62	11.34	5.76	0.004
60 days	6.55	4.66	10.95	4.77	0.009
